# Increased DCLK1 correlates with the malignant status and poor outcome in malignant tumors: a meta-analysis

**DOI:** 10.18632/oncotarget.20129

**Published:** 2017-08-10

**Authors:** Wenhua Shi, Fangwei Li, Shaojun Li, Jian Wang, Qingting Wang, Xin Yan, Qianqian Zhang, Limin Chai, Manxiang Li

**Affiliations:** ^1^ Department of Respiratory and Critical Care Medicine, The First Affiliated Hospital of Xi’an Jiaotong University, Xi’an, Shaanxi 710061, China

**Keywords:** DCLK1, tumors, biomarker, diagnosis, meta-analysis

## Abstract

Doublecortin-like kinase 1 (DCLK1) has been found to be involved in malignant biological behavior of cancers and poor prognosis of cancer patients. The aim of this meta-analysis was to systematically clarify the relationships between expression level of DCLK1 and clinicopathological characteristics in tumors and assess its clinical value in cancer diagnosis and prognosis. 18 eligible studies with a total of 2660 patients were identified by searching the electronic bibliographic databases. Pooled results showed that DCLK1 was highly expressed in tissues from cancer patients compared to normal tissues (OR, 10.00), and overexpression of DCLK1 was significantly correlated with advanced clinical stage (OR, 2.48), positive lymph node metastasis (OR, 2.18), poorly differentiated cancers (OR, 1.83) and poor overall survival (HR, 2.15). The overall combined sensitivity and specificity for DCLK1 in distinguishing malignant tumors were 0.58 and 0.90, respectively. The mean diagnostic odds ratio was 12.70, and the corresponding area under the summary receiver operating characteristic curve was 0.78. In summary, our study indicated that DCLK1 could be a risk factor for development of malignant tumors and may serve as a promising diagnostic and prognostic biomarker for malignant tumors.

## INTRODUCTION

Cancer is one of the leading causes of mortality and is a major public health problem worldwide. It has been reported that 1,688,780 new cancer cases and 600,920 cancer deaths are projected to occur in the United States in 2017 [1]. Despite medical and scientific efforts over the past decades, patients with malignant tumors often face poor clinical outcomes [2]. It has been demonstrated that the early diagnosis and reliable prediction for recurrences are critical for the prognosis of cancer patients [3]. Of note, biomarkers are already important adjuvant tools for refining and optimizing diagnosis, treatment, and prognosis [4]. However, currently established biomarkers, due to limited validation and questionable prognostic values, could not qualify as reliable biomarkers for early diagnosis and prognosis assessment in clinical practice [5]. Therefore, it will be of enormous importance to identify new markers to help diagnose tumors, predict clinical outcomes and serve as therapeutic targets.

Doublecortin-like kinase 1 (DCLK1) is a microtubule-associated protein that catalyzes the polymerization of tubulin dimmers and the formation of aster-like microtubule structures [6]. DCLK1 has been identified to be involved in tumorigenesis of various types of cancer, such as renal cell carcinoma (RCC) [7], pancreatic ductal adenocarcinoma (PDAC) [8], colorectal cancer (CRC) [9] and breast carcinoma (BCA) [10]. In addition, it has been demonstrated that DCLK1 is associated with malignant biological behavior and poor prognosis of cancer [11, 12]. Knockdown of DCLK1 has been shown to significantly reduce invasion, migration and focal adhesion of RCC cells, indicating that DCLK1 may be a potential therapeutic target for RCC [7]. Although quite a number of studies have suggested that DCLK1 could be a risk factor and prognostic biomarker for malignant tumors, the relationships between expression level of DCLK1 and clinicopathological characteristics in tumors, especially for the clinical stage, lymph node metastasis and the degree of tumor differentiation, are still largely unknown and deserve further research. Therefore, we conducted the comprehensive meta-analysis on all eligible studies to clarify these and assess the clinical value of DCLK1 in cancer diagnosis and prognosis, thereby providing more evidence for clinical practice and accelerating further investigations.

## RESULTS

### Searching process of literature

**Figure 1:**
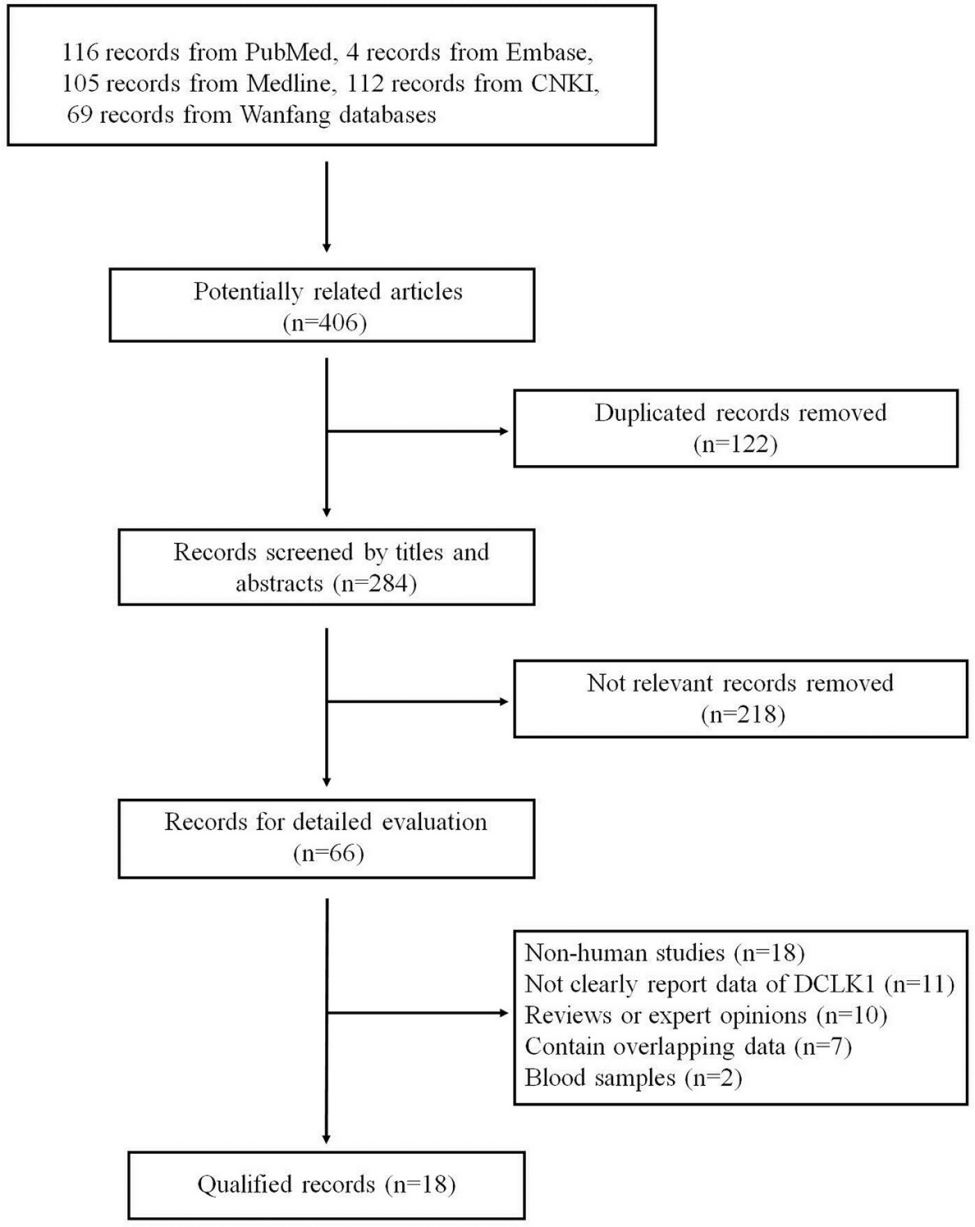
The flow diagram of study selection for meta-analysis

The complete literature search yielded 406 articles: 116 from PubMed, 105 from Medline, 4 from Embase, 112 from Chinese National Knowledge Infrastructure (CNKI) and 69 from Wanfang databases. And 284 articles were screened based on titles and abstracts after removing 122 duplicates. Among these articles, 266 articles were excluded for the following reasons: obviously not relevant to the topic (*n =* 218), non-human studies (*n =* 18), not clearly report data of DCLK1 (*n =* 11), reviews or expert opinions (*n =* 10), contain overlapping data (*n =* 7), serum samples (*n =* 2). Finally, 18 articles were included in this meta-analysis. Figure 1 is a flow diagram outlining the study-selection process.

### Study characteristics

As shown in Table 1, 18 studies [8–25] investigated the association between DCLK1 expression and clinicopathological characteristics in patients with cancer from 2012 to 2017, containing a total of 2660 patients with the sizes of studies distributing from 23 [18] to 1132 [10] patients. All studies were retrospective, and most of studies adopted immunohistochemistry (IHC) to detect the expression of DCLK1 in cancer tissues samples that composed of malignant cell and a supporting stroma, while one adopted reverse transcription-polymerase chain reaction (RT-PCR). The source of the malignant tumor included PDAC [8], CRC [9, 16, 17, 19, 22, 23], BCA [10, 15], head and neck squamous cell carcinoma (HNSCC) [11], non-small cell lung cancer (NSCLC) [12], oral squamous-cell carcinoma (OSCC) [13], gastric cancer (GC) [14, 20], hepatocellular carcinoma (HCC) [18], salivary gland carcinoma (SGC) [21], malignant pleural mesothelioma (MPM) [24] and bladder cancer (BC) [25]. In these studies, most of investigations were in Asia, including China and Japan. Four studies were in America [8, 18, 23, 24], and two studies were in Austria [11, 21].

**Table 1:** Main characteristics of the 18 studies included in the meta-analysis

Source	Author	Year	Country	Enrolled period	Research design	Resources of samples	Test method	Cancer /control	Cancer		Tumor stage (N)		Tumor differentiation(N)		Lymphatic Metastasis (N)	
									Age	M/F(n)	I–II	III–IV	Well to moderate	Poor	Yes	No
CRC	Tianbo Gao [9]	2016	China	2007 to 2011	Retrospective	Tumor tissue	IHC	71/16	60	44/27	28	43	61	10	41	30
	Huan Wang [16]	2015	China	2007to 2012	Retrospective	Tumor tissue	IHC	150/20	58.4	91/59	-	-	95	36	66	84
	Anjun Le [19]	2015	China	2007 to 2008	Retrospective	Tumor tissue	IHC	70/70	52.6 ± 10.5	42/28	-	-	21	49	36	34
	Shuxiang An [17]	2015	China	2009 to 2013	Retrospective	Tumor tissue	IHC	60/20	-	38/22	34	26	48	12	25	35
	Malaney R O’Connell [22]	2015	Japan	2005 to 2011	Retrospective	Tumor tissue	RT-PCR	92/0	68	57/35	49	43	82	10	41	51
	Giuseppe Gagliardi [23]	2012	USA	2000 to 2010	Retrospective	Tumor tissue	IHC	40/0	66	23/17	14	26	26	14	-	-
GC	Lin Chen [14]	2015	China	2013.3 to 2013.10	Retrospective	Tumor tissue	IHC	49/49	28～70	27/22	19	30	22	27	36	13
	Qingbin Meng [20]	2013	China	2002 to 2006	Retrospective	Tumor tissue	IHC	122/122	62	86/36	-	-	-	-	85	37
BCA	Jingjing Gan [15]	2016	China	2005 to 2007	Retrospective	Tumor tissue	IHC	129/129	53	0/129	56	73	86	43	94	35
	Yuhong Liu [10]	2015	China	2002 to 2009	Retrospective	Tumor tissue	IHC	1132/0	54.6 ± 12.7	0/1132	-	-	630	502	542	557
NSCLC	Hiroyuki Tao [12]	2017	Japan	2005 to 2009	Retrospective	Tumor tissue	IHC	232/0	61	128/104	232	-	-	-	39	193
HCC	Sripathi M. Sureban [18]	2015	USA	2000 to 2010	Retrospective	Tumor tissue	IHC	23/23	62 ± 13.8	10/13	4	18	-	-	11	12
SGC	Lorenz Kadletz [21]	2017	Austria	1970 to 2013	Retrospective	Tumor tissue	IHC	80/0	58	43/37	41	39	-	-	58	22
HNSCC	Lorenz Kadletz [11]	2017	Austria	2002 to 2012	Retrospective	Tumor tissue	IHC	127/0	57.7	-	20	107	-	-	99	28
OCCC	Xin Wu [13]	2017	China	2013 to 2014	Retrospective	Tumor tissue	IHC	30/30	-	-	-	-	-	-	-	-
PDAC	Dongfeng Qu [8]	2015	USA	-	Retrospective	Tumor tissue	IHC	12/62	64	35/27	31	31	-	-	-	-
MPM	Hui Wang [24]	2017	USA	1997 to 2008	Retrospective	Tumor tissue	IHC	73/8	68.13	-	15	17	-	-	-	-
BC	Shiqing Zhang [25]	2017	China	2005 to 2015	Retrospective	Tumor tissue	IHC	118/40	-	79/39	49	69	80	38	16	102

CRC, colorectal cancer; GC, gastric cancer; BCA, breast carcinoma; NSCLC, non-small cell lung cancer; HCC, hepatocellular carcinoma; SGC, salivary gland carcinoma; HNSCC, head and neck squamous cell carcinoma; OSCC, oral squamous-cell carcinoma; PDAC, pancreatic ductal adenocarcinoma; MPM, malignant pleural mesothelioma; BC, bladder cancer; IHC, immunohistochemistry, RT-PCR, real time polymerase chain reaction.

### Cancer group vs. control group

**Figure 2:**
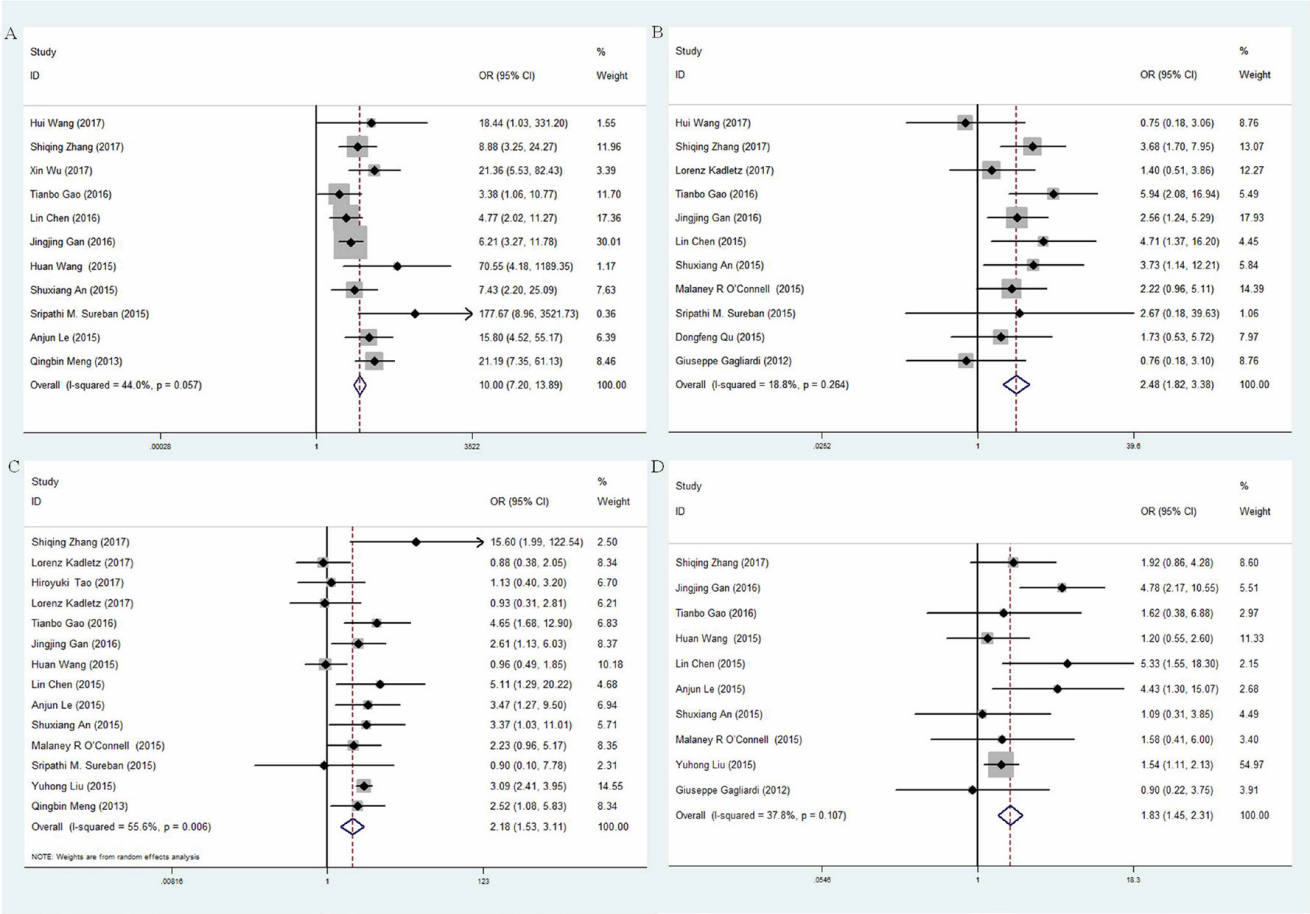
Forest plot of odd ratio (OR) of subgroup analysis (**A**) subgroup analysis based on control tissues; (**B**) subgroup analysis based on TNM stage; (**C**) subgroup analysis based on lymphatic metastasis; (**D**) subgroup analysis based on differentiation.

Eleven studies investigated the expression patterns of DCLK1 in various cancer and normal tissues [9, 13–20, 24, 25], which included 742 cancer cases and 508 normal controls (Table 2). The overall odds ratio (OR) was 10.00 (95% CI = 7.20–13.89) (Z = 13.75, *p <* 0.001) (Figure 2A). Subgroup analysis was stratified according to geographic region. The summary OR in Asian region was 9.25 (95% CI = 6.61–2.94, *P <* 0.001) and 48.61 (95% CI = 5.89–401.25, *P <* 0.001) in non-Asia region (Table 3). Significant association existed between DCLK1 level and cancer tissues, indicating that expression of DCLK1 was dramatically higher in cancer tissues than that in normal tissues.

**Table 2:** DCLK1 expression in control and cancer patients

Author			Expression of DCLK1 (positive/all) (*n*)						Diagnostic test				
	Control (*n*)	Cancer ((*n*)	Tumor stage (*n*)		Tumor differentiation (*n*)		Lymphatic metastasis(n)		TP	FP	FN	TN	OS HR (95% CI) (U)
			I–II	III–IV	Well tomoderate	Poor	Yes	No					
Tianbo Gao [9]	5/16	43/71	10/28	33/43	36/61	7/10	31/41	12/30	43	5	28	11	-
Huan Wang [16]	0/20	95/150	-	-	38/95	16/36	26/66	34/84	95	0	55	20	-
Anjun Le [19]	3/70	29/70	-	-	4/21	25/49	20/36	9/34	29	3	41	67	-
Shuxiang An [17]	4/20	39/60	18/34	21/26	23/48	6/12	20/25	19/35	39	4	21	16	-
Malaney R O’Connell [22]	-	46/92	20/49	26/43	40/82	6/10	25/41	21/51	-	-	-	-	3.55 (1.41–8.99)
Giuseppe Gagliardi [23]	-	27/40	10/14	17/26	8/26	4/14	-	-	-	-	-	-	4.16 (1.28–13.57)
Lin Chen [14]	18/49	36/49	7/19	22/30	6/22	18/27	25/36	4/13	36	18	13	31	-
Qingbin Meng [20]	4/122	51/122	-	-	-	-	41/85	10/37	51	4	71	118	2.27 (1. 36–3.80)
Jingjing Gan [15]	15/129	58/129	18/56	40/73	28/86	30/43	48/94	10/35	58	15	71	114	2.12 (1.24–3.71)
Yuhong Liu [10]	-	418/1132	-	-	277/630	141/502	178/542	222/557	-	-	-	-	-
Hiroyuki Tao [12]	-	33/232	33/232	-	-	-	5/39	28/193	-	-	-	-	1.80 (1.13–2.85)
Sripathi M. Sureban [18]	0/20	19/23	3/4	16/18	-	-	9/11	10/12	15	1	8	19	-
Lorenz Kadletz [21]	-	53/80	9/41	11/39	-	-	15/58	6/22	-	-	-	-	-
Lorenz Kadletz [11]	-	66/127	-	-	-	-	50/99	15/28	-	-	-	-	2.00 (1.20–3.40)
Xin Wu [13]	4/30	23/30	-	-	-	-	-	-	23	4	7	26	-
Dongfeng Qu [8]	-	23/44	10/22	13/22	-	-	-	-	-	-	-	-	-
Hui Wang [24]	0/8	37/73	9/15	9/17	-	-	-	-	38	35	0	8	-
Shiqing Zhang [25]	5/40	65/118	18/49	47/69	40/80	25/38	15/16	50/102	66	52	5	35	3.35 (2.01–5.6)

*n,* cases; TP, true positive; FP, false positive; FN, false negative; TN, true negative; OS, overall survival; HR, hazard ratio; U, univariate analysis

**Table 3:** Subgroup analysis of study region

Subgroup	Region	Study (*n*) number	Patients (*n*)	Controls (*n*)	OR/HR	(95% Conf.Interval)	*Z*	*P*	I^2^
	overall	11	742	508	10.00	(7.20–13.89)	13.75	< 0.001	44.00%
**Cancer vs. control**	Asia	9	649	460	9.25	(6.61–12.94)	12.99	< 0.001	39.90%
	non-Asia	2	96	48	48.61	(5.89–401.25)	3.61	< 0.001	13.50%
	overall	11	380	266	2.48	(1.82–3.38)	5.78	< 0.001	18.80%
**TNM stage**	Asia	6	284	188	3.29	(2.28–4.76)	6.34	< 0.001	0.00%
	non-Asia	5	96	78	1.21	(0.67–2.19)	3.02	< 0.001	0.00%
	overall	14	1172	1199	2.18	(1.53–3.11)	4.31	< 0.001	55.60%
**Lymph node metastasis**	Asia	11	1013	1155	2.76	(2.29–3.33)	10.58	< 0.001	46.50%
	non-Asia	3	159	44	0.90	(0.48–1.71)	0.32	> 0.05	0.00%
	overall	10	389	1411	1.83	(1.45–2.31)	5.06	< 0.001	37.80%
**Differentiation degree**	Asia	9	375	1385	1.87	(1.47–2.37)	5.16	< 0.001	40.80%
	non-Asia	1	14	26	0.90	(0.22–3.75)	0.14	> 0.05	-
	overall	7	860	291	2.15	(1.64–2.65)	8.33	< 0.001	0.00%
**OS**	Asia	5	693	-	2.17	(1.60–2.74)	7.44	< 0.001	0.00%
	non-Asia	2	167	-	2.07	(0.98–3.15)	3.74	< 0.001	0.00%

OS, overall survival; OR, odds ratio; HR, hazard ratio.

### Clinical stages of cancers: advanced stage vs. early stage

Eleven studies [8, 9, 14, 15, 17, 18, 21–25] investigated the expressions of DCLK1 in different clinical stages of cancers. Patients with cancer were divided into early stage (I-II stage) and advanced stage (III–IV stage) based on the tumor-node-metastasis (TNM) classification: 266 cancer cases of early stage and 380 cases of advanced stage (Table 2). The overall OR was 2.48 (95% CI = 1.82–3.38) (Z = 5.78, *p <* 0.001) via a fixed model analysis (Figure 2B). Subgroup analysis stratified by study region showed that the summary OR was 1.21 (95% CI = 0.67–2.19, *P <* 0.001) in non-Asia countries while was 3.29 (95% 95% CI = 2.28–4.76, *P <* 0.001) in Asian countries with significant association (Table 3), suggesting that expression level of DCLK1 was dramatically higher in advanced stage group than that in early stage group.

### Lymph node metastasis of cancers: positive group vs. negative group

Fourteen studies [9–12, 14–22, 25] investigated the expressions of DCLK1 in different status of lymphatic metastasis, including 1172 cases of positive lymphatic metastasis and 1199 cases of negative lymphatic metastasis (Table 2). The overall OR was 2.18 (95% CI = 1.53-3.11) (*Z* = 4.31, *p <* 0.001), indicating that expression level of DCLK1 was markedly higher in positive group than that in negative group. We used a random-effect model justified by the high heterogeneity (I^2^ = 55.60%, *p* = 0.006) (Figure 2C). Subgroup analysis stratified by study region showed that the summary OR of Asian studies was 2.76 (95% CI = 2.29–3.33, *p <* 0.001) with significant association. However, the statistic significance in non-Asia region disappeared (OR = 0.9, 95% CI = 0.48-1.71, *p* > 0.05) (Table 3).

### Degree of tumor differentiation: poor group vs. well and moderate group

As shown in Table 2, ten studies [9, 10, 14–17, 19, 22, 23, 25] investigated the expressions of DCLK1 in different degree of tumor differentiation, including 1411 cases of well and moderate differentiation and 389 cases of poor differentiation. The overall OR was 1.83 (95% CI = 1.45-2.31) (Z = 5.06, *p <* 0.001), indicating that expression level of DCLK1 was relatively higher in the poor group than that in the well and moderate group (Figure 2D). Subgroup analysis stratified by study region showed that the summary OR in Asian region was 1.87 (95% CI = 1.47-2.37, *p <* 0.001) and 0.9 (95% CI = 0.22–3.75, *P* > 0.05) in non-Asia region (Table 3). Significant association only existed in Asia.

### Correlation between DCLK1 expression and overall survival (OS)

**Figure 3:**
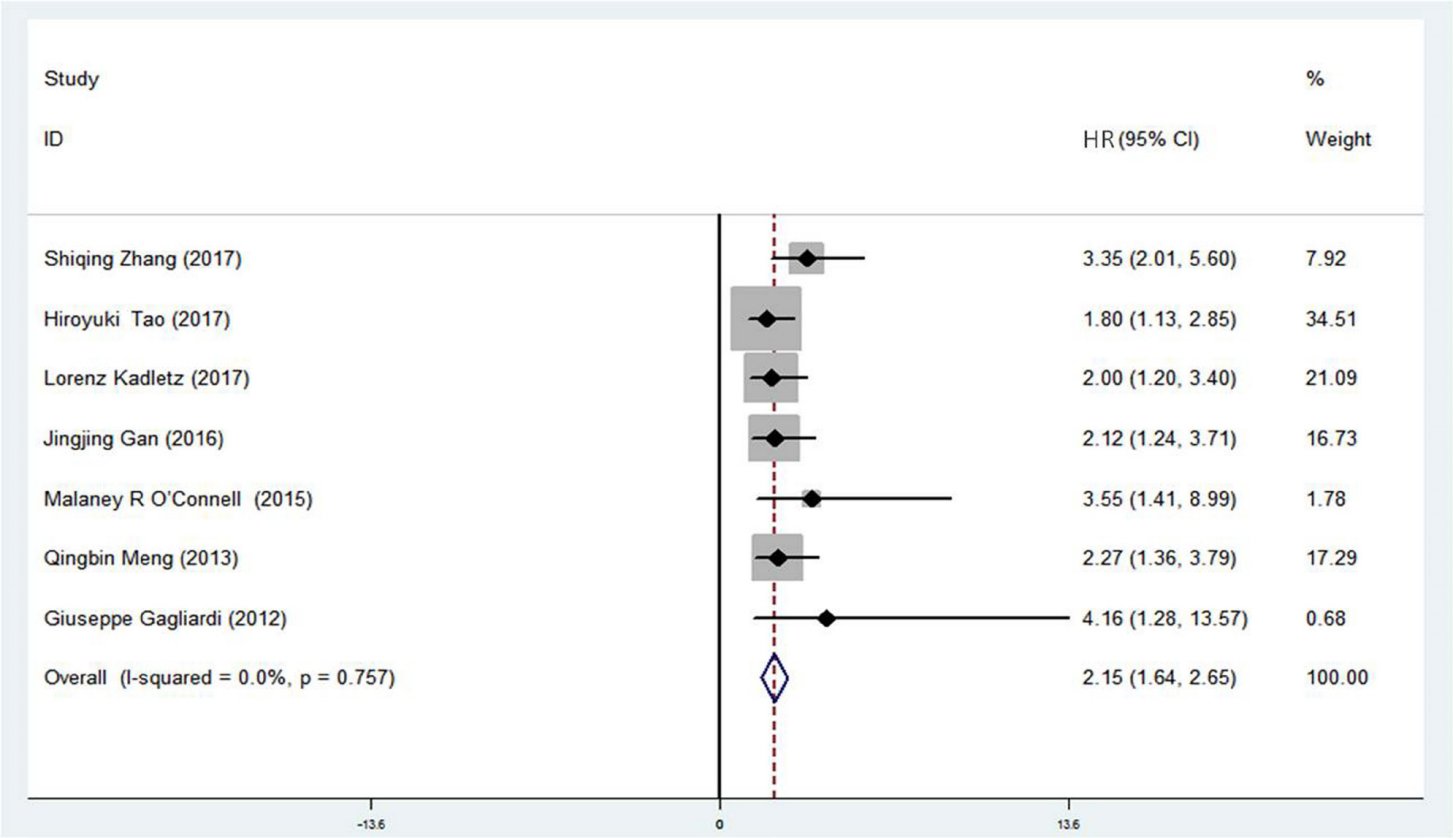
Association between DCLK1 and overall survival for patients with cancer

In order to investigate the association between DCLK1 expression and clinical outcome in malignant tumors, patients were divided into DCLK1-high and DCLK1-low groups. Based on the results of univariate analysis in the original studies, a total of 7 pairs of hazard ratio (HR) for OS were available in the 18 studies [11, 12, 15, 20, 22, 23, 25] (Table 2). The combined HR was 2.15 (95% CI = 1.64–2.65) (Z = 8.33, *p <* 0.001) (Figure 3). Subgroup analysis stratified by study region showed that the summary HR in Asian region was 2.17 (95% CI = 1.60-2.74, *p <* 0.001) and 2.07 (95% CI = 0.98–3.15, *p <* 0.001) in non-Asia region. Significant association existed between DCLK1 level and OS (Table 3), indicating that DCLK1 may act as a potential marker for predicting survival outcomes in patients with cancer.

### Accuracy of DCLK1 for diagnosing cancers

**Figure 4:**
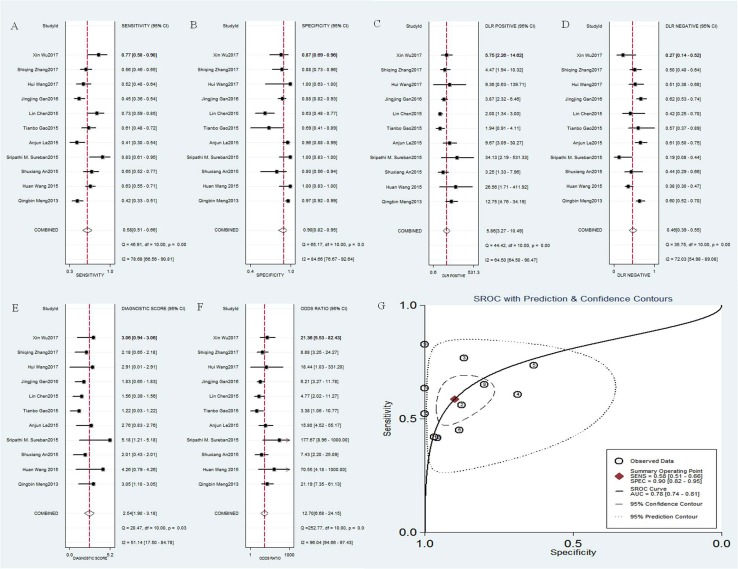
Diagnostic accuracy of DCLK1 in cancer (**A**) sensitivity of DCLK1 for the diagnosis of cancer; (**B**) specificity of DCLK1 for the diagnosis of cancer; (**C**) positive likelihood ratio (PLR) of DCLK1 for the diagnosis of cancer; (**D**) negative likelihood ratio (NLR) of DCLK1 for the diagnosis of cancer; (**E**) diagnostic score of DCLK1 for the diagnosis of cancer; (**F**) diagnostic odds ratio (DOR) of DCLK1 for the diagnosis of cancer; (**G**) the corresponding area under the SROC curve (AUC) of DCLK1 for the diagnosis of cancer.

A total of 11 studies reported sensitivity and specificity for distinguishing cancers and normal tissues [9, 13–20, 24, 25]. The computation of Spearman correlation coefficient between the logit of sensitivity and logit of 1-specificity showed no evidence of a threshold effect (Spearman correlation coefficient = –0.58; p = 0.34). The overall combined sensitivity, specificity, positive likelihood ratio (PLR), negative likelihood ratio (NLR), diagnostic score and relevant 95% CI were as follows: 0.58 (95% CI = 0.51-0.66) (Figure 4A), 0.90 (95% CI = 0.82–0.95) (Figure 4B), 5.9 (95% CI = 3.3–10.5) (Figure 4C), 0.46 (95% CI = 0.39–0.55) (Figure 4D) and 2.54 (95% CI = 1.90-3.18) (Figure 4E), respectively. Significant heterogeneity was observed for sensitivity (I^2^ = 78.68%, *p <* 0.001), specificity (I^2^ = 84.66%, *p <* 0.001), PLR (I^2^ = 64.50%, *p <* 0.001), NLR (I^2^ = 72.03 %, *p <* 0.001) and diagnostic score (I^2^ = 51.14 %, *p <* 0.001). The mean diagnostic odds ratio (DOR) was 12.70 (95% CI = 6.68–24.15) (Figure 4F), indicating that DCLK1 level in cancer tissues could be helpful in the diagnosis of malignant tumors. The summary receiver operating characteristic (SROC) curve analysis was used to analyze the effectiveness of DCLK1 for diagnostic purposes, the corresponding area under the SROC curve (AUC) was 0.78 (Figure 4G), revealing moderate diagnostic accuracy overall.

### Meta-regression and subgroup analysis

**Figure 5:**
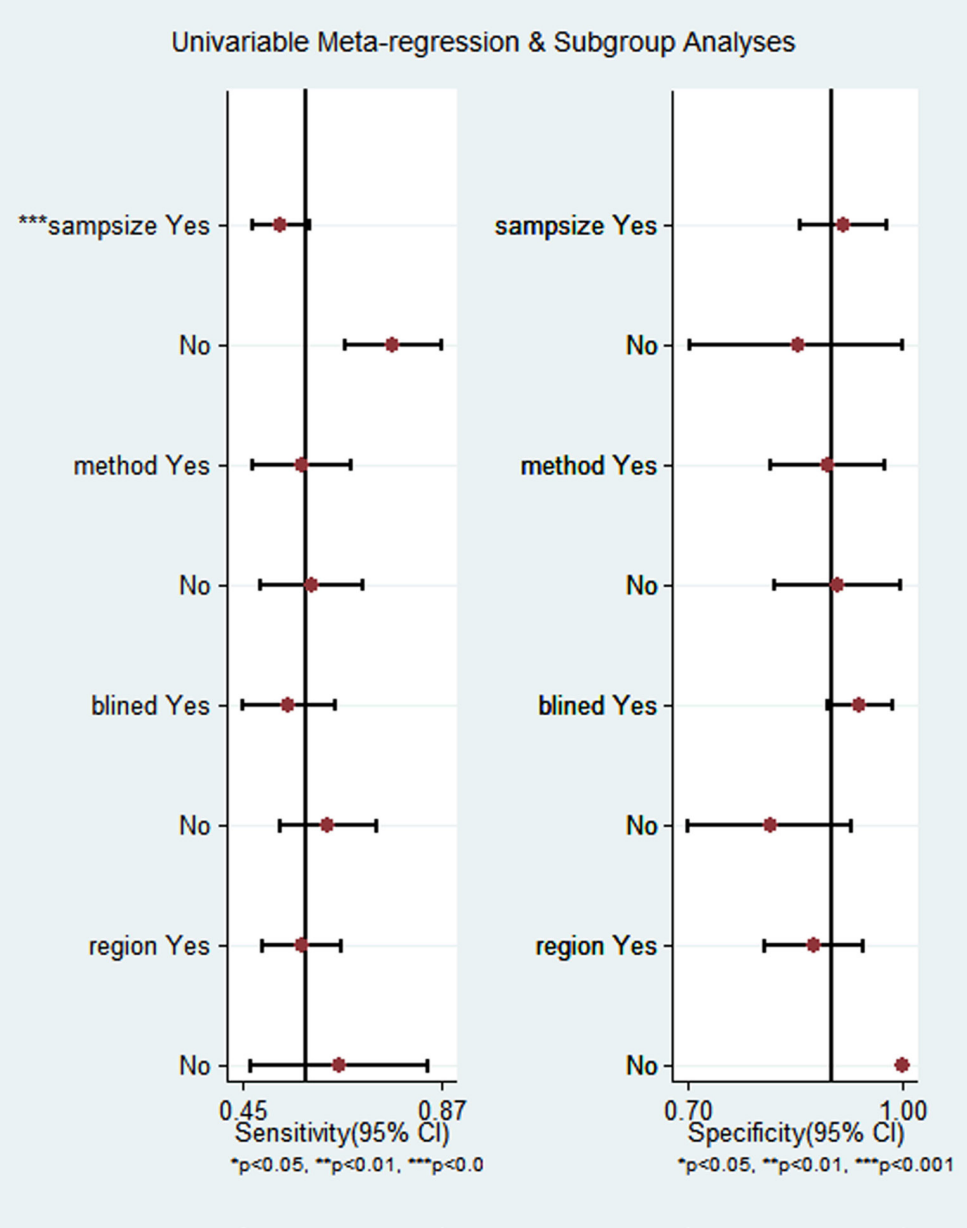
Univariable meta-regression and subgroup analysis

To explore the sources of high heterogeneity in sensitivity and specificity, we performed univariate meta-regression and subgroup analysis according to sample size, study design, detection method and geographic region. As shown in Figure 5, sample size may be a significant impact factor for the high heterogeneity in this meta-analysis. In studies with more than 50 subjects, the pooled sensitivity, specificity, PLR, NLR, DOR, AUC of DCLK1 were 0.62 (95% CI = 0.44–0.77), 0.85 (95% CI = 0.67–0.94), 4.1 (95% CI = 2.1–7.9), 0.45 (95% CI = 0.32–0.62), 9 (95% CI = 5–116) and 0.79, respectively. And those for studies with fewer subjects were 0.76 (95% CI = 0.67–0.84), 0.78 (95% CI = 0.68-0.86), 4.78 (95% CI = 1.21–18.88), 0.30 (95% CI = 0.20-0.47), 17 (95% CI = 3–93) and 0.81, respectively, which indicated a comparable diagnostic accuracy between the large- and small-sample studies. Similar results were found in the subgroup analyses according to study design, detection method and geographic region (Table 4).

**Table 4:** Summarized results of diagnostic criteria

Subgroup	Category	Study number	Patients(n)	Controls(n)	Sensitivity	Specificity	PLR	NLR	DOR	AUC
**All combined**	Overall	11	674	488	0.58 (0.51–0.66)	0.90 (0.82–0.95)	5.9 (3.3–10.5)	0.46 (0.39–0.55)	13 (7–24)	0.78
**Sample size**	≥ 50	8	572	389	0.62 (0.44–0.77)	0.85 (0.67–0.94)	4.1 (2.1–7.9)	0.45 (0.32–0.62)	9 (5–116)	0.79
	> 50	3	102	99	0.76 (0.67–0.84)	0.78 (0.68–0.86)	4.78 (1.21–18.88)	0.30 (0.20–0.47)	17 (3–93)	0.81
**Blinded**	Yes	6	475	361	0.55 (0.44–0.67)	0.95 (0.87–0.98)	10.9 (3.9–30.2)	0.47 (0.36–0.62)	23 (7–76)	0.85
	No	5	199	127	0.78 (0.52–0.92)	0.67 (0.40–0.86)	2.4(1.4–4.1)	0.33 (0.18–0.62)	7 (4–14)	0.79
**Method**	Multiplication	5	335	261	0.58 (0.44–0.72)	0.90 (0.73–0.97)	6.0 (2.1–16.8)	0.46 (0.34–0.63)	13 (4–39)	0.77
	Addition	6	339	227	0.75 (0.44–0.92)	0.78 (0.54–0.92)	3.5 (1.9–6.4)	0.32 (0.14–0.72)	11 (6–22)	0.84
**Region**	Asia	9	614	424	0.58 (0.49–0.65)	0.88 (0.79–0.93)	4.6 (2.9–7.5)	0.48 (0.42–0.56)	10 (6–16)	0.76
	non-Asia	2	60	64	0.93 (0.84–0.98)	0.44 (0.31–0.57)	6.06 (0.00–7848)	0.18 (0.08–0.39)	54 (5–530)	–

PLR, positive likelihood ratio; NLR, negative likelihood ratio; DOR, diagnostic odds ratio; AUC, corresponding area under the summary receiver operating characteristic curve.

### Quality assessment of studies

**Figure 6:**
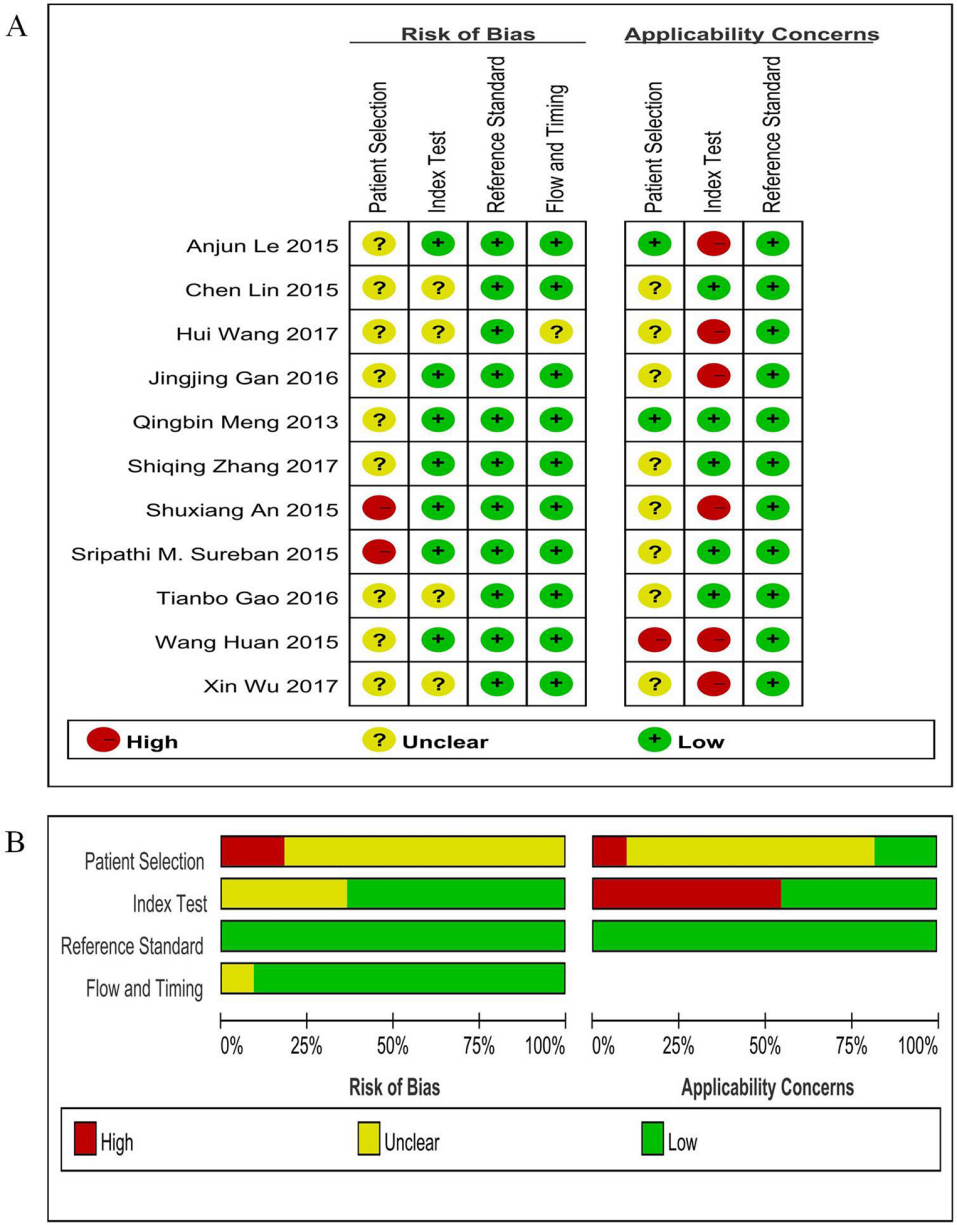
Assessment of methodological quality of diagnostic accuracy studies (**A**) risk of bias and applicability concerns summary; (**B**) risk of bias and applicability concerns graph.

Two reviewers evaluated the quality of each study independently, most of 18 studies had more than 5 stars of scores based on Newcastle-Ottawa Scale (NOS) (Supplementary Table 1). Figure 6A and 6B showed the results of study methodological quality evaluation according to Quality Assessment of Diagnostic Accuracy Studies (QUADAS-2) items, suggesting that the overall quality of included studies was moderate to high.

### Publication bias and sensitivity analysis

The funnel plot was used to evaluate publication bias, and the shape of the funnel appeared to be approximately symmetrical. Furthermore, no significant publication bias was detected by Egger’s test and Deeks’ funnel plot (Supplementary Figures 1 and 2). The stability of the results was detected by sensitivity analysis, we found that exclusion of individual studies has no effect on the overall results (Supplementary Figure 3).

## DISCUSSION

In this meta-analysis including 18 retrospective studies with a total of 2660 patients, we clarified the association between expression of DCLK1 and the clinicopathological characteristics of malignant tumors. Our results indicated that the expression of DCLK1 in cancer tissues was significantly higher than that in normal or adjacent non-tumor tissues. After implementing a series of subgroup analysis, we noticed that high expression of DCLK1 was significantly correlated with poorly differentiated cancers, positive lymph node metastasis and advanced clinical stage, suggesting that overexpression of DCLK1 markedly accelerated the pathogenesis and development of cancer. Furthermore, we also found that patients with high DCLK1 expression had significantly poor overall survival, indicating that DCLK1 may be a promising biomarker that helps to identify patients with a high risk for recurrence in cancer. In subgroup analysis stratified by geographic region (Asia vs non-Asia), overexpression of DCLK1 was significantly correlated with advanced clinical stage and poor overall survival for all people, and non-significant heterogeneity was found (I^2^ = 0.00%). However, high DCLK1 expression was only notably correlated with positive lymph node metastasis and poorly differentiated cancer in Asian region but not in non-Asia region.

Additionally, we assessed the diagnostic accuracy of DCLK1 in malignant tumors. The overall pooled sensitivity and specificity were 0.58 (95% CI = 0.51–0.66) and 0.90 (95% CI = 0.82–0.95), respectively, which suggested that DCLK1 had a relatively considerable accuracy in detecting malignant tumors. The PLR of 5.9 (95% CI = 3.3–10.5) suggested that patients with cancer have a 5.9-fold higher chance of being DCLK1 test positive compared to individuals without cancer. Similarly, the NLR of 0.46 (95% CI = 0.39–0.55) indicated that low expression of DCLK1 might help exclude non-cancer individuals. These ratios suggested a potential role of DCLK1 for clinical confirmation and exclusion purpose. The DOR is the ratio of the odds of positivity in patients relative to the odds of positivity in control subjects, with higher values indicating better discriminatory test performance. In our meta-analysis, the value of DOR was 13, indicating that DCLK1 could be used as a biomarker for the diagnosis of malignant tumors. When sensitivity and specificity were considered simultaneously, the AUC of DCLK1 was 0.78, suggesting that the test performance of DCLK1 in discerning cancer tissues is reasonably good.

Studies have shown that DCLK1 negatively regulates tumor suppressor miRNAs and promotes epithelial-mesenchymal transition (EMT), a process through which polarized epithelial cells undergo multiple biochemical changes, leading to a mesenchymal phenotype, such as enhanced migratory capacity, invasiveness, and resistance to apoptosis [9, 18, 26]. Furthermore, DCLK1 has been shown to decrease tumor suppressor miRNAs let-7a, miR-200, miR-144*,* and miR-143/145 and subsequently up-regulate their downstream targets, including c-MYC, EMT-associated transcription factors ZEB1, ZEB2, Snail and Slug in HCC, CRC and PDAC, thereby promoting the development of cancer [9, 18, 27]. In addition, DCLK1 also regulates Notch-1 via a miR-144 dependent mechanism and its downstream effector HES1 to promote tumor xenograft growth [28]. Knockdown of DCLK1 or application of small molecule kinase inhibitors for DCLK1 could result in a delay of tumor development by down-regulating miRNAs downstream pluripotency transcription factors, EMT and critical oncogenic pathways [18, 26, 27, 29]. Recently, it has been reported that combination of DCLK1 inhibition with irradiation has a synergistic effect in HNSCC cell lines [11]. DCLK1 is therefore considered as an attractive and potential therapeutic target in the treatment of malignant tumors [30, 31]. Due to the limitations of currently available evidence on the therapeutic effects and molecular biology of DCLK1 in malignant tumors, Further studies are warranted to investigate these in more detail.

There was significant heterogeneity exiting across the included studies, which is a potential obstacle. Firstly, the heterogeneity was probably due to differences in cancer types included in this meta-analysis. Secondly, variable cut-off points were used in different studies, which may lead to the threshold effect contributing to heterogeneity, even calculated Spearman correlation coefficient value was –0.58 (*p* = 0.34). In addition, we found DCLK1 has higher diagnostic accuracy for malignant tumors in small samples using univariate meta-regression analysis and subgroup analysis, suggesting that sample size may be partially the cause of heterogeneity in sensitivity and specificity.

Though it was the first meta-analysis to assess the diagnostic and prognostic role of DCLK1 in cancer, there were still several limitations in our study. First, the majority of the studies in this meta-analysis were retrospective investigations conducted in Asia, which may result in potential selection bias. Second, despite a systematic and exhaustive literature search was performed, the sample sizes and number of included studies were relatively small, which may place restrictions on evaluating the diagnostic performance of DCLK1. Third, we could not determine the optimal cut-off value for DCLK1 due to use of different cut-off values for the examined sample set.

Despite these limitations, the meta-analysis still provided credible evidence that up-regulation of DCLK1 was an early event in the carcinogenesis and progression of malignant tumors. Expression level of DCLK1 was closely correlated to tumor differentiation, lymph node invasion and clinical phase of the patients. It was useful for predicting development of malignant tumors and clinical outcome of cancer patients. In addition, DCLK1 was potential to be a promising diagnostic and prognostic biomarker for malignant tumors, and might serve as an attractive therapeutic target in the treatment of malignant tumors.

## MATERIALS AND METHODS

### Search strategy

Two independent reviewers performed a comprehensive and systematic literature search to identify relevant studies from the databases of PubMed, Medline, Embase, Chinese National Knowledge Infrastructure (CNKI) and Wanfang databases (from inception through July 2017). The search strategy used following free text and Medical Subject Heading terms to increase sensitivity. The search strings was: (“cancer” or “tumor” or “carcinoma” or “tumors” or “neoplasm” or “malignant neoplasm” or “malignant tumors”) and (“Doublecortin and CaM kinase-like-1” or “Doublecortin-like kinase 1” or “DCLK1” or “KIAA0369”). All searches were restricted to English and Chinese publications. The reference lists of relevant articles were manually searched.

### Inclusion and exclusion criteria

The inclusion criteria: (1) sufficient data on DCLK1 expression in malignant tumors; (2) case-control, cross-sectional, or cohort studies; (3) inclusion of pathologically confirmed patients; (4) patient tissues were taken prior to chemotherapy, radiotherapy and other drug treatment. The exclusion criteria: (1) reviews, editorials, expert opinions and case reports; (2) no useful data reported; (3) non-human studies; (4) unqualified or did not provide sufficient data.

### Data extraction and quality assessment of studies

We carried out this meta-analysis in accordance with PRISMA guidelines [32]. The following types of characteristics were extracted: study characteristics (the name of first author, publication year, country, enrolled period, study design and testing method of DCLK1), patient characteristics (age, sex, numbers of patients, case number of different groups, histological classification, tumor differentiation degree, tumor node metastases, TNM classification, HR and 95% CI), and concrete data of DCLK1 expression (number of true positives, false negatives, false positives, and true negatives). We systematically assessed the quality of primary studies using the Newcastle-Ottawa Scale (NOS) [33], and assessed the methodological quality based on the QUADAS-2 list to ensure that the enrolled studies were accurate and reliable [34]. Any disagreements were discussed and resolved by consensus.

### Statistical analysis

After sufficient data were collected and identified, we calculated the pooled OR and 95% CI using two different meta-analysis approaches according to heterogeneity. The chi-squared test and *I*^2^ test were used to evaluate the heterogeneity of the studies and considered significant at *p <* 0.1 or I^2^ > 50% [35]. We calculated the effect sizes using fixed effects models with low heterogeneity (I^2^ < 50% or *p* > 0.1). Otherwise, a random-effects model was presented [36]. If necessary, we performed meta-regression and subgroup analysis to explore potential sources of heterogeneity. Pooled sensitivity, specificity, PLR, NLR, DOR and their 95%CI were displayed as forest plots [37]. In addition, we extracted the HR and 95%CI from the papers to perform meta-analysis of HR to determine the association of DCLK1 expression with survival of patients. Funnel plots, Egger’s test and Deeks’ funnel plot were implemented to measure potential publication bias, *p* ≥ 0.05 indicates no publication bias [38, 39], and sensitivity analysis was performed to evaluate whether exclusion of any individual study affects the overall results. All statistical analyses were performed using STATA software (Version 12.0; STATA Corporation, College Station, TX). Two-tailed *p* values < 0.05 were considered statistically significant (except for the heterogeneity and publication bias tests already mentioned).

## SUPPLEMENTARY MATERIALS FIGURES AND TABLE



## Abbreviations

CRC: colorectal cancer
GC: gastric cancer
BCA: breast carcinoma
NSCLC: non-small cell lung cancer
HCC: hepatocellular carcinoma
SGC: salivary gland carcinoma
HNSCC: head and neck squamous cell carcinoma
OSCC: oral squamous-cell carcinoma
PDAC: pancreatic ductal adenocarcinoma
MPM: malignant pleural mesothelioma
BC: bladder cancer
IHC: Immunohistochemistry
RT-PCR: real time polymerase chain reaction
TP: true positive
FP: false positive
FN: false negative
TN: true negative
OS: overall survival
HR: hazard ratio
U: univariate analysis
DCLK1: Doublecortin-like kinase 1
OR: odds ratio
PLR: positive likelihood ratio
NLR: negative likelihood ratio
DOR: diagnostic odds ratio
SROC: summary receiver operating characteristic
