# Supplementary material for: Increased DCLK1 correlates with the malignant status and poor outcome in malignant tumors: a meta-analysis
Source: Oncotarget. 2017 Aug 10;8(59):100545–57. doi: 10.18632/oncotarget.20129 (PMC5725042; doi:10.18632/oncotarget.20129)
Supplement: Supplementary file 1 [file oncotarget-08-100545-s001.pdf]

Increased DCLK1 correlates with the malignant status and poor outcome in malignant tumors: a meta-analysis

SUPPLEMENTARY MATERIALS

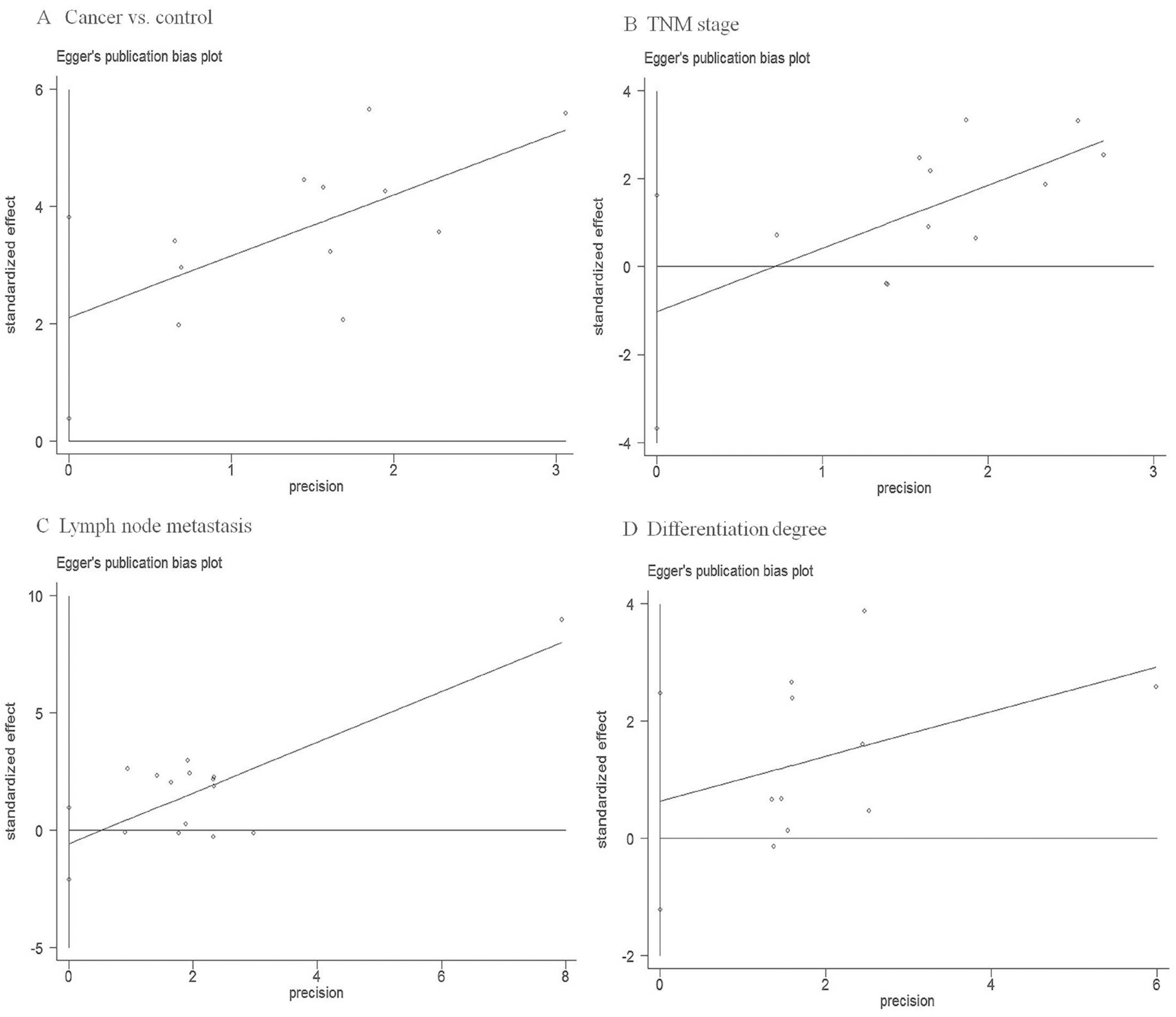

Supplementary Figure 1: Egger's test of potential publication bias of the included studies for the correlation between DCLK1 expression and clinicopathological characteristics in malignant tumors.

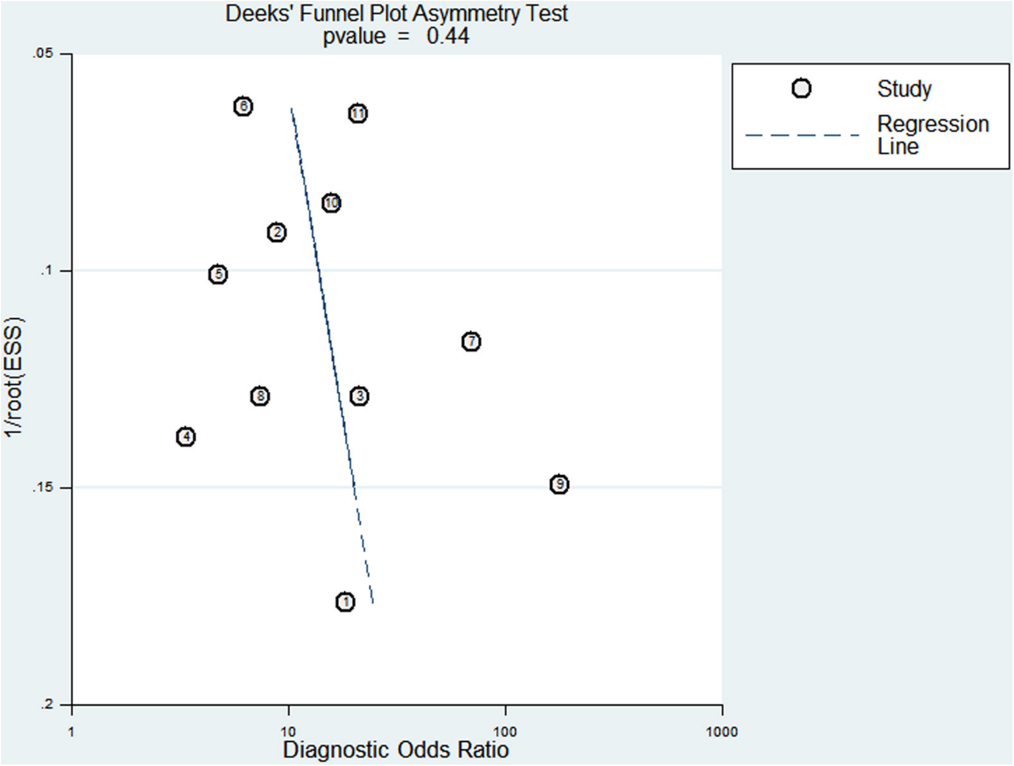

Supplementary Figure 2: Deeks' funnel plot asymmetry test for publication bias.

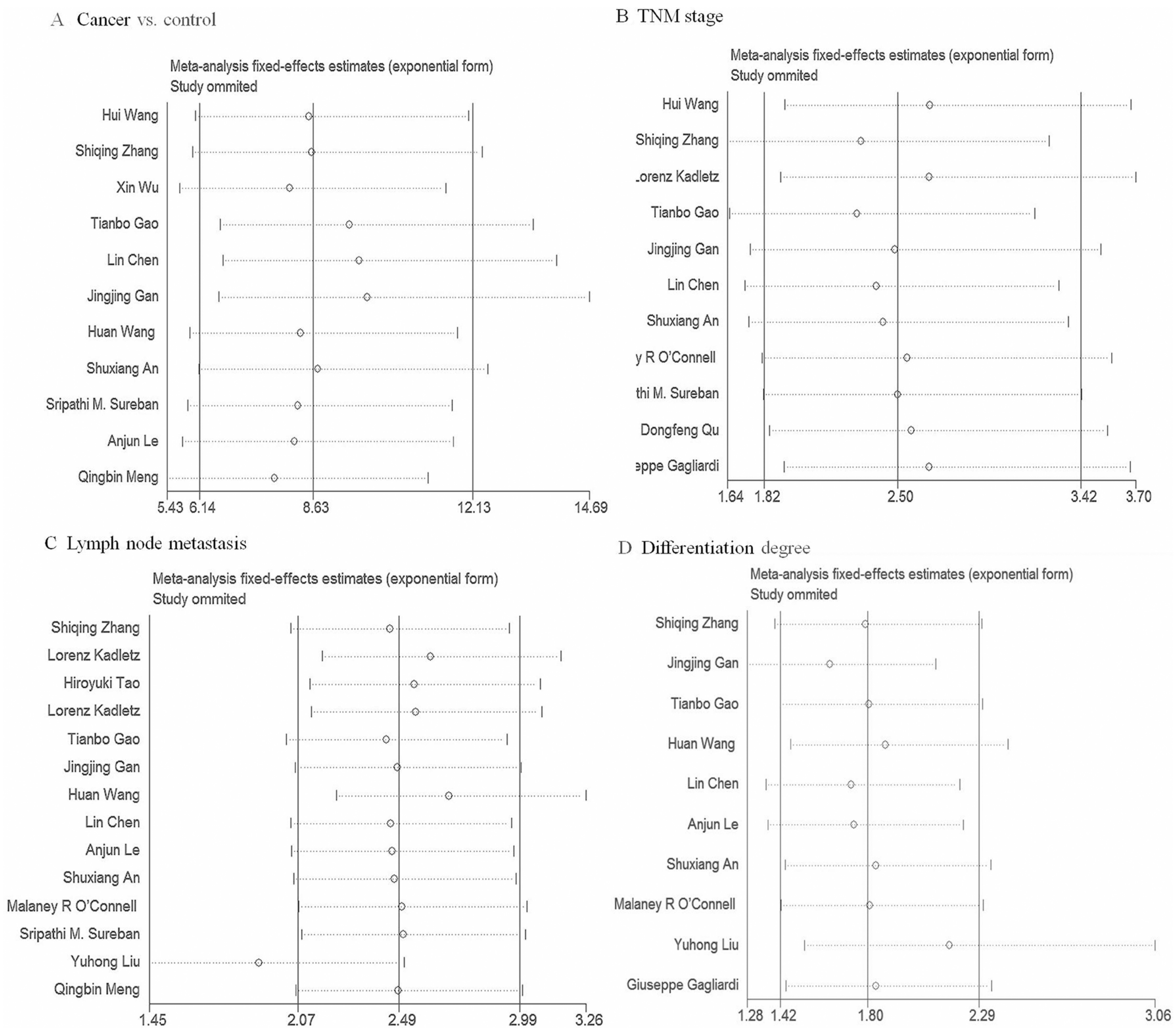

Supplementary Figure 3: Summary of sensitivity analysis of the included studies for the correlation between DCLK1 expression and clinicopathological characteristics in malignant tumors.

Supplementary Table 1: Assessing the quality of included studies using the Newcastle-Ottawa Scale (NOS)

Author	Q1	Q2	Q3	Q4	Q5	Q6	Q7	Q8	Q9	Star
Tianbo Gao [9]				☆	☆		☆	☆		4
Wang Huan [16]	☆	☆	☆	☆		☆	☆	☆		7
Anjun Le [19]	☆	☆		☆	☆		☆	☆		6
Shuxiang An [17]	☆	☆		☆		☆	☆	☆		6
Malaney R O'Connell [22]	☆	☆					☆		☆	4
Giuseppe Gagliardi [23]	☆	☆				☆	☆	☆		5
Lin Chen [14]	☆	☆		☆	☆		☆	☆		6
Qingbin Meng [20]	☆	☆				☆	☆	☆		5
Jingjing Gan [15]	☆	☆		☆		☆	☆	☆		6
Yuhong Liu [10]	☆	☆				☆	☆	☆		5
Hiroyuki Tao [12]	☆	☆				☆	☆	☆		5
Sripathi M. Sureban [18]	☆	☆	☆	☆	☆	☆	☆	☆		8
Lorenz Kadletz [21]	☆	☆					☆	☆	☆	5
Lorenz Kadletz [11]	☆	☆			☆		☆	☆		5
Xin Wu [13]	☆	☆			☆	☆	☆	☆		6
Dongfeng Qu [8]	☆	☆		☆		☆	☆	☆		6
Hui Wang [24]	☆	☆		☆			☆	☆		5
Shiqing Zhang [25]	☆	☆		☆		☆	☆	☆	☆	6

Question 1: Is the case definition adequate?.

Question 2: Representativeness of the cases.

Question 3: Selection of Controls.

Question 4: Definition of Controls.

Question 5: Comparability of cases.

Question 6: Controls on the basis of the design or analysis.

Question 7: Ascertainment of exposure.

Question 8: Same method of ascertainment for cases and controls.

Question 9: Non-response rate.
